# Noninvasive Two-Phase Foaling Prediction in Thoroughbred Mares Using Thermal Imaging and AI-Based Behavioral Analysis

**DOI:** 10.3390/ani16142221

**Published:** 2026-07-17

**Authors:** Hisashi Nabenishi, Nagisa Taki, Shoji Nishibayashi, Tomoyuki Ishii

**Affiliations:** 1 Laboratory of Animal Feeding and Management, Department of Animal Science, School of Veterinary Medicine, Kitasato University, Higashi 23-35-1, Aomori 034-8628, Japan; 2 Noritsu Precision Co., Ltd., Wakayama 640-8550, Japan

**Keywords:** equine reproduction, parturition surveillance, computer vision, precision livestock farming, thoroughbred mare, noninvasive monitoring, foaling detection

## Abstract

Early and accurate detection of foaling is important for reducing perinatal mortality and labor demands in commercial breeding farms. This study evaluated a fully noninvasive foaling prediction system integrating thermal imaging with artificial intelligence-based behavioral analysis in Thoroughbred mares. A total of 115 pregnant mares across 13 farms were monitored during the 2024 foaling season. Locomotor activity and body surface temperature relative to ambient temperature were extracted from thermal images, while posture changes and tail-raising behavior were identified from visible-light images using a trained prediction model. Prediction was performed at 5 min intervals using a pretrained neural network model implemented in Python, which generated logistic probabilities based on locomotor activity, body surface temperature, posture change frequency, and tail-raising behavior. Data obtained during the 5 h preceding foaling were analyzed. Locomotor activity and surface temperature significantly increased 70–90 min before foaling (*p* < 0.05), whereas posture changes and tail-raising behavior markedly increased 25–45 min before foaling. These temporal patterns suggest a biologically interpretable two-phase pre-foaling process derived from sequential changes in behavioral and thermal indicators. The model based on locomotor activity and surface temperature achieved an 80.0% detection rate with a mean lead time of 186 ± 20 min. Incorporating posture changes and tail-raising parameters improved detection to 94.8% and reduced the detection-to-foaling interval to 89 ± 14 min. The system achieved high predictive performance under commercial conditions without requiring invasive or wearable devices.

## Simple Summary

Foaling often occurs quickly and frequently at night, and delayed assistance can threaten both the mare and newborn foal. Many breeding farms rely on continuous human observation or invasive devices to detect the onset of birth, increasing labor demands and potentially causing stress. In this study, we evaluated a completely non-contact camera system to predict foaling in Thoroughbred mares under commercial farm conditions. The system continuously monitored body movement, body surface temperature, posture changes between standing and lying, and tail-raising behavior in 115 mares across 13 farms. Movement and surface temperature increased approximately 1–1.5 h before foaling, whereas clear posture and tail changes appeared closer to delivery. By combining these early and late signs, the system correctly predicted foaling in 94.8% of mares. This noninvasive approach enables continuous monitoring without attaching devices to animals and may help reduce nighttime labor while improving foaling safety and animal welfare.

## 1. Introduction

Efficient reproductive management is critical to the economic sustainability of Thoroughbred breeding operations. The average length of gestation of mares is approximately 340 days; however, individual variation commonly exceeds 20 days and may range from 320 days to over 360 days [1,2,3]. This wide variability renders prediction based solely on the breeding date unreliable. Furthermore, approximately 70–80% of foaling occurs during nighttime—an evolutionary adaptation believed to reduce the risk of predation [4,5]. Consequently, breeding farms must maintain continuous surveillance for extended periods, imposing substantial labor, economic, and psychological burdens on personnel.

Early assistance during foaling is important because dystocia, although relatively uncommon in mares compared with cattle, progresses rapidly and may result in severe consequences for both mare and foal [6,7,8]. Among Thoroughbreds, perinatal mortality remains a significant concern, with prompt obstetrical intervention being a decisive factor [9,10]. Therefore, accurately predicting the timing of parturition has direct implications for animal welfare, foal survival, and farm productivity.

Traditional foaling prediction relies on external physical signs, including the enlargement of mammary glands, waxing of teats, vulvar relaxation, softening of the pelvic ligaments, and changes in milk electrolyte concentration or Brix values [11,12,13,14]. The Brix refractometry method estimates total dissolved solids in mammary secretions and has been used as an indirect indicator of impending parturition [14]. Although these indicators provide useful guidance, they exhibit marked interindividual variability and are influenced by individual and management-related factors. Moreover, many of these parameters require repeated manual handling, thereby increasing labor and potentially inducing stress.

Therefore, technological approaches have been developed to improve prediction accuracy. Vaginal temperature loggers and intravaginal transmitters detect expulsion at membrane rupture and can provide highly accurate alerts; nevertheless, they are invasive and require careful placement and hygiene management [15,16,17]. Vulvar magnetic sensors can detect the separation of sutured magnets at foaling onset, but they require surgical suturing and may cause discomfort or local complications [18,19]. Wearable accelerometers have been used to detect restlessness and rolling behavior; however, these devices can be displaced, and their battery life limits long-term monitoring [20,21,22]. However, despite these technological advances, important limitations remain. Invasive devices require placement and periodic maintenance, which may increase handling stress and the risk of device displacement. Wearable sensors can also be detached, require battery management, and may generate false alerts due to non-parturition-related activity. In addition, many existing systems monitor a single physiological or behavioral indicator, which may reduce robustness under variable farm conditions. Therefore, a fully noninvasive system capable of integrating multiple complementary indicators without animal attachment is still needed for practical and reliable foaling prediction under commercial breeding conditions.

Recent advances in computer vision and deep learning have enabled continuous, non-contact monitoring of animal behavior in livestock systems [23,24,25]. In horses, image-based monitoring has been explored for lameness detection, behavior classification, and stall activity tracking [26,27,28]. In addition, thermal imaging has been used to assess peripheral circulation, inflammation, and stress responses in equine subjects [29,30,31]. However, no studies have comprehensively integrated behavioral metrics and thermal data for predictive foaling detection under commercial breeding conditions.

Previous studies have reported increased locomotor activity, restlessness, frequent lying and standing, and tail-raising behavior immediately before foaling [32,33,34]. In parallel, subtle increases in body temperature have been documented during the periparturient period, reflecting hormonal and metabolic changes [35,36]. Nevertheless, most studies examined these parameters independently and in small experimental cohorts. Large-scale validation under real farm conditions, particularly in Thoroughbred populations, remains limited.

In addition, equine behavior is strongly influenced by daily activity rhythms and management-related time budgets. Deviations from baseline behavioral allocation, including locomotion, resting, and posture transitions, may therefore provide early indicators of physiological state changes. Recent work has demonstrated that time–activity budgets in horses are sensitive to both management conditions and physiological transitions and can serve as robust indicators of impending events such as parturition. Integrating such behavior-based indicators with physiological signals may improve the reliability of foaling prediction under practical farm conditions [37,38].

Recent studies have explored automated foaling detection using image-based monitoring and deep-learning approaches. Camera-based behavioral analysis has been applied to identify prepartum behavioral changes and estimate the onset of foaling in mares [39]. Deep-learning-based systems using RGB and thermal imaging have also been proposed for automated detection of foaling-related events, demonstrating the feasibility of noncontact monitoring [40]. Furthermore, convolutional neural network-based object detection and behavioral classification approaches have been developed for automatic identification of parturition-related behaviors in horses [41]. However, most of these studies were conducted using limited datasets or single-farm conditions, and large-scale validation under commercial Thoroughbred breeding environments remains limited.

Therefore, the primary objective of this study was to develop and evaluate a noninvasive foaling prediction model integrating behavioral and thermal indicators. The secondary objectives were to characterize temporal changes in pre-foaling parameters, examine undetected cases, and interpret the results within a biologically meaningful two-phase prepartum framework.

## 2. Materials and Methods

### 2.1. Animals and Management

In this study, 115 pregnant Thoroughbred mares housed in 13 commercial breeding farms in Hokkaido, Japan, were enrolled during the foaling season from January to April 2024. The mean age of mares was 11.3 ± 4.3 years (range: 5–23 years). The mean parity of the mares was 3.7 ± 2.8 (SD), ranging from 1 to 12. No cases of dystocia were recorded during the study period. All mares were clinically healthy and managed in accordance with each farm’s standard husbandry practices. They were fed roughage and concentrate feeds formulated to meet the nutritional requirements for maintenance and late pregnancy. Meals were provided daily in the morning and late afternoon. Fresh water and mineralized rock salt were available ad libitum. All mares were pastured daily between 07:00 and 15:00 and individually housed in box stalls between 15:00 and 07:00. Behavioral and thermal monitoring was exclusively performed during the stall-housing period. Stall dimensions varied among farms but were typically approximately 3.5 m × 3.5 m to 4.5 m × 4.5 m, providing sufficient space for normal prepartum movement while allowing the entire stall area to be captured within the camera field of view. The stalls’ dimensions and bedding materials followed each farm’s conventional management practices. All procedures were observational and noninvasive. No experimental interventions were applied to the animals.

### 2.2. Monitoring System

The mares were continuously monitored using a digital camera system (Umamori, Noritsu Precision Co., Ltd., Wakayama, Japan), as described previously [40]. The system comprised a thermal infrared imaging camera integrated with a visible-light camera, enabling simultaneous acquisition of thermal and RGB images. The camera unit was mounted on the stall wall at approximately 2.5 m above the ground and positioned to capture the entire stall area from a diagonal upper angle, ensuring that the mare’s whole body remained within the field of view.

Thermal and visible images were recorded continuously. Locomotor activity was calculated from thermal images by separating the mare from the background using binarization, followed by automatic centroid detection and trajectory analysis. The distance moved was computed from successive centroid coordinates using the Euclidean distance between two points. Movement was calculated in two-dimensional image space based on centroid displacement. Although depth information was not included because of the diagonal camera placement, the fixed camera geometry allowed consistent relative estimation of locomotor activity within each stall. Therefore, the calculated distance was interpreted as a relative activity index rather than absolute movement distance.

Body surface temperature was measured at 1 s intervals from thermal images. Temperature was calculated as the mean value of the segmented mare region, and minimum or maximum temperature points were not used. Ambient temperature was simultaneously recorded, and the difference between body surface temperature and ambient temperature was calculated to minimize environmental effects. Because whole-body segmentation was applied, positional variation in specific body parts relative to the camera had limited influence on the averaged temperature value. Visible images were simultaneously used for model-based detection of posture changes and tail-raising behavior. All data were automatically stored and processed for behavioral and thermal analysis. For the present analysis, data obtained during the 5 h preceding foaling were extracted and used for statistical evaluation and algorithm validation.

### 2.3. Parameter Extraction and Machine-Learning-Based Probability Models

Two prediction models were evaluated using a machine-learning-based probability framework implemented in the Umamori system. Two models were evaluated to assess stepwise integration of previously reported prepartum indicators. Model 1 included locomotor activity and body surface temperature, which have been reported as early physiological and behavioral changes preceding foaling. Model 2 additionally incorporated posture change frequency and tail-raising behavior, representing late-stage behavioral signs closer to expulsion. This stepwise design allowed evaluation of incremental improvement in prediction accuracy. The prediction model was constructed using a neural network implemented in Python 3.10 and was previously trained using an independent dataset consisting of 53 foaling events reported previously [40]. The present study applied the pretrained model without additional training or parameter tuning. Two models were evaluated to assess the contribution of late-phase behavioral indicators. Model 1 included locomotor activity and body surface temperature as early prepartum indicators, whereas Model 2 additionally incorporated posture change frequency and tail-raising behavior.

Prediction was performed at 5 min intervals, and each interval was treated as an independent prediction unit. In Model 1, locomotor activity and body surface temperature relative to ambient temperature were used as input variables. In Model 2, posture change frequency and tail-raising behavior were added, resulting in four input variables. Locomotor activity was expressed in pixel units derived from centroid displacement in thermal images. This pixel-based measurement reflects relative movement within the camera field of view and provides a consistent index of locomotor activity without requiring spatial calibration.

The model output was expressed as the logistic probability of imminent foaling. The prediction model was trained using an independent dataset reported previously [40], and the present dataset was not used for model training or parameter tuning. Therefore, images and behavioral data from the current experiment were used solely for evaluation. No data supplementation, retraining, or exposure of evaluated foaling events to the model was performed. Because the model had been trained using independent data, the present dataset was used solely for evaluation of predictive performance. No additional training, data partitioning, or cross-validation was performed in the current dataset.

The probability threshold for foaling detection was set at 70%, which was adopted as a default value based on prior experimental data reported previously [40]. This threshold was selected before the present analysis and was not optimized using the current dataset. The use of a predefined threshold allowed consistent evaluation of detection performance while minimizing the risk of overfitting to the present study population. Although statistical analysis focused on the 5 h period preceding foaling, prediction probabilities were calculated continuously throughout the monitoring period. Therefore, notifications could occur earlier than the 5 h analysis window when probability values exceeded the predefined threshold.

### 2.4. Statistical Analysis

All statistical analyses were performed using JMP 13.2.0 software (SAS Institute Inc., Cary, NC, USA). Differences were considered statistically significant at *p* < 0.05. Data obtained 300 min (5 h) before foaling were defined as the control reference point for time-course analysis of prepartum changes. Each subsequent time interval was compared with this control value using Dunnett’s test to adjust for multiple comparisons against a single control. Because repeated measurements were obtained from the same mares, the temporal data were not fully independent; therefore, the statistical analysis was primarily descriptive and intended to identify general temporal trends. Data are presented as the mean ± standard error (SE).

Because the analysis aimed to identify general temporal trends, formal tests of normality and homogeneity of variance were not performed prior to applying parametric comparisons. The results should therefore be interpreted as descriptive comparisons, and more advanced modeling approaches may be considered in future studies.

## 3. Results

### 3.1. Prepartum Changes in Behavioral and Thermal Parameters

**Figure 1:**
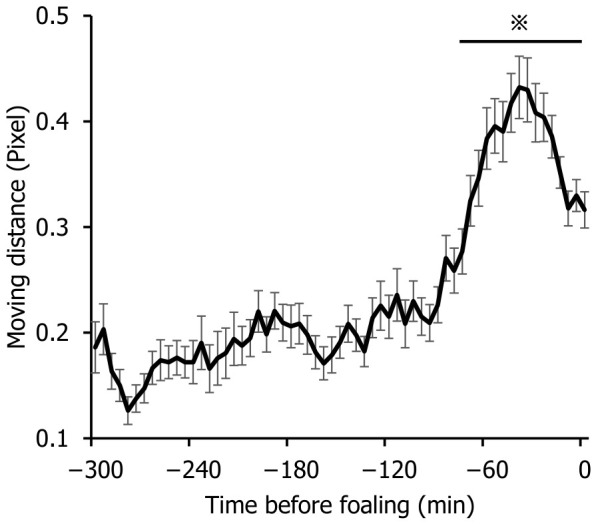
Temporal changes in locomotor activity during the 5 h preceding foaling. Data are presented as the mean ± standard error (SE). Time 0 represents the moment of foaling. Meanwhile, asterisks indicate significant differences compared with the value at −300 min (*p* < 0.05). Locomotor activity was expressed as moving distance in pixel units derived from centroid displacement in thermal images. Pixel-based movement represents relative displacement within the image frame and provides a consistent index of locomotor activity across individuals.

**Figure 2:**
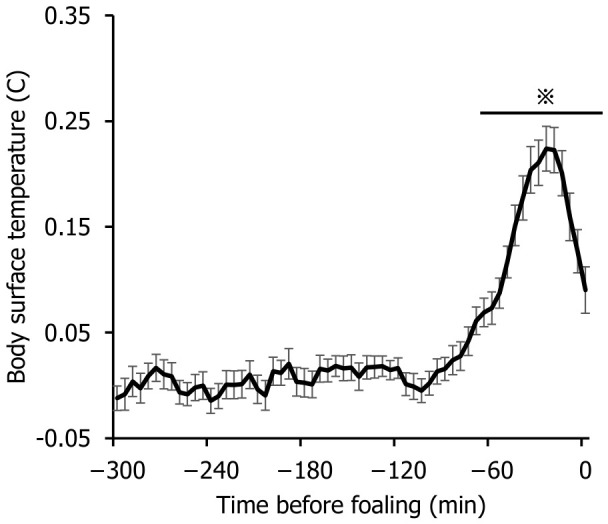
Temporal changes in surface temperature measured by thermal imaging during the 5 h preceding foaling. Values represent the mean body surface temperature difference between the segmented mare region and ambient temperature. Data are presented as the mean ± SE. Time 0 represents the moment of foaling. Meanwhile, asterisks indicate significant differences compared with the value at −300 min (*p* < 0.05).

Locomotor activity increased significantly beginning 70 min before foaling compared with 300 min pre-foaling (*p* < 0.05), increasing from 0.19 ± 0.02 to 0.32 ± 0.02. Surface body temperature difference also increased from −0.01 ± 0.01 to 0.06 ± 0.01 during the same period. Posture change frequency increased significantly from 25 min before foaling, rising from 0.06 ± 0.04 to 0.73 ± 0.15 (*p* < 0.05), whereas tail-raising behavior increased from 45 min pre-foaling, from 0.64 ± 0.15 to 1.73 ± 0.20 (*p* < 0.05) (Figure 1 and Figure 2).

**Figure 3:**
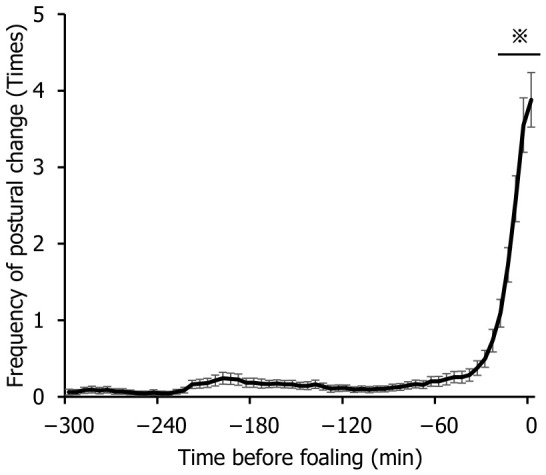
Temporal changes in posture change frequency during the 5 h preceding foaling. Data are presented as the mean ± SE. Time 0 represents the moment of foaling. Meanwhile, asterisks indicate significant differences compared with the value at −300 min (*p* < 0.05).

**Figure 4:**
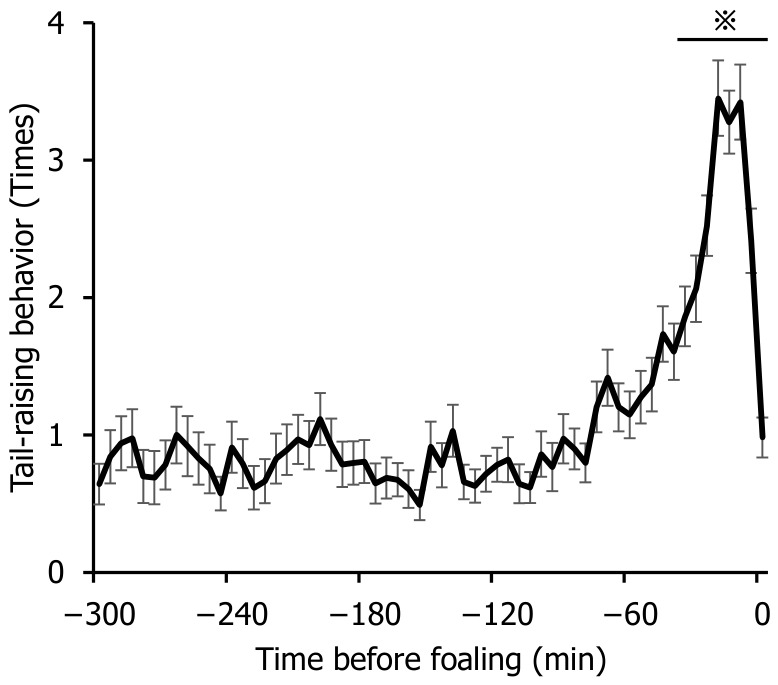
Temporal changes in tail-raising behavior during the 5 h preceding foaling. Data are presented as the mean ± SE. Time 0 represents the moment of foaling. Meanwhile, asterisks indicate significant differences compared with the value at −300 min (*p* < 0.05).

The posture change frequency and tail-raising behavior also increased as parturition approached. Posture changes significantly increased from 25 min before foaling (*p* < 0.05; Figure 3), whereas tail-raising behavior increased from 45 min pre-foaling (*p* < 0.05; Figure 4). The temporal comparison of parameter dynamics revealed distinct pre-foaling patterns. Locomotor activity and surface temperature began increasing approximately 90 min before foaling, whereas posture change and tail-raising behavior increased more prominently from approximately 30 min before foaling. Based on these findings, changes in thermal and locomotor parameters preceded the more overt behavioral changes observed immediately before foaling.

### 3.2. Comparison of Foaling Detection Performance Between Model 1 and Model 2

The foaling detection performance differed between the two predictive models (Table 1). A total of 92 out of 115 mares were detected prior to foaling using locomotor activity and surface temperature as predictive indicators (Model 1), corresponding to a detection rate of 80.0%. The mean interval from first detection to foaling was 186 ± 20 min. When posture change frequency and tail-raising behavior were incorporated into the machine-learning-based probability model (Model 2), 109 mares were detected prior to foaling, yielding a detection rate of 94.8%. The mean detection-to-foaling interval decreased to 89 ± 14 min. Thus, the inclusion of late-phase behavioral parameters increased detection sensitivity and improved temporal proximity to foaling.

Using detection prior to foaling as the criterion for successful prediction, the sensitivity of Model 2 was 94.8% (109/115). Prediction probabilities were calculated continuously throughout the monitoring period, whereas statistical analysis focused on the 5 h interval preceding foaling. Pre-foaling alerts were generated in 32 mares (27.8%) two days before foaling and 47 mares (40.9%) one day before foaling, with mean notification counts of 1.02 ± 0.25 and 2.00 ± 0.38, respectively. The earliest notification occurred two days before foaling. These early alerts occurred outside the 5 h analysis window, whereas the mean number of alerts increased to 4.82 ± 0.39 on the day of foaling. Early notifications observed two and one day before foaling were generally isolated and associated with gradual increases in predicted probability rather than sustained high-probability states. In most cases, the prediction probability increased intermittently during the prepartum period and rose sharply on the day of foaling, resulting in sustained alerts close to delivery.

**Figure 5:**
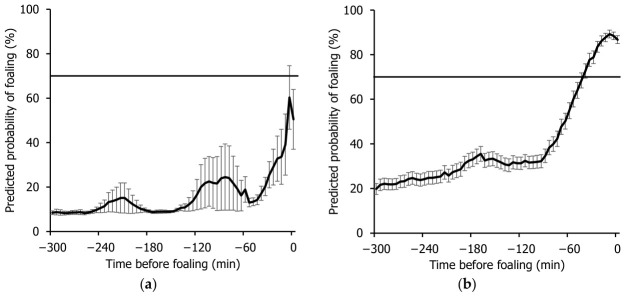
Temporal changes in the predicted foaling probability using model 2 during the 5 h preceding foaling. (**a**) Predicted foaling probability in the four mares that were not detected prior to foaling. In these cases, the probability gradually increased but did not exceed the predefined detection threshold of 70%. (**b**) Predicted foaling probability in the 109 mares that were successfully detected before foaling. In all detected cases, the probability exceeded the 70% threshold prior to foaling. Time 0 represents the moment of foaling. Meanwhile, the dashed horizontal line indicates the predefined detection threshold (70%).

Four mares were not detected prior to foaling. No clear differences were observed in age or parity between detected and undetected mares. In these cases, locomotor activity showed minimal increases, resulting in predicted probabilities that did not exceed the predefined 70% threshold. These findings suggest that some mares exhibit only subtle behavioral changes prior to foaling (Figure 5).

## 4. Discussion

This study represents the first large-scale multi-farm validation of a fully noninvasive foaling prediction system integrating thermal imaging and behavioral analysis in Thoroughbred mares. The integrated system achieved a detection rate of 94.8% under commercial breeding conditions, a performance that favorably compares with wearable accelerometer systems (approximately 70–90%) [22,23] and approaches the reported accuracy of invasive vaginal transmitter devices without the associated handling stress or risk of premature expulsion [17,18,19]. Importantly, this study not only demonstrates the system’s high predictive accuracy but also provides mechanistic insights into the sequential physiological and behavioral processes preceding foaling.

### 4.1. Biological Interpretation of the Two-Phase Prepartum Pattern

The present study provides a noninvasive multimodal approach integrating thermal and behavioral indicators and evaluates its performance across multiple commercial breeding farms. These variables were selected based on previously reported prepartum behavioral and physiological changes and are interpreted here in relation to the biological stages of parturition. The present findings suggest two temporally distinct prepartum phases. Locomotor activity and surface temperature increased approximately 70–90 min before delivery, whereas posture change frequency and tail-raising behavior increased markedly within 25–45 min before foaling. The early rise in locomotor activity may reflect restlessness associated with uterine contractions, abdominal discomfort, and endocrine activation during stage I labor [34,35,36]. The concurrent elevation in surface temperature difference may also reflect peripheral vasodilation and increased muscular activity during early parturition [35,36]. In contrast, the abrupt increase in posture transitions and tail elevation appeared closer to delivery and may correspond to the transition from stage I to stage II labor [6,7]. These findings are consistent with previous descriptions of frequent standing–recumbency transitions and repetitive tail lifting during the expulsive phase [8,34]. Taken together, the temporal separation observed in this study supports a biologically interpretable two-phase framework; however, this interpretation remains inferential and requires further physiological validation.

### 4.2. Enhanced Detection Performance Through Structured Integration

Model 1, which incorporated locomotor activity and surface temperature, achieved a detection rate of 80.0% with a mean lead time of approximately 3 h. Although this provided a useful preparatory warning, temporal dispersion was relatively broad. The integration of posture change frequency and tail-raising behavior (model 2) increased the detection sensitivity to 94.8% and reduced the detection-to-foaling interval to 89 ± 14 min. This improvement reflects the complementary predictive roles of early- and late-phase indicators. Early-phase parameters enhance sensitivity by detecting initial physiological activation, whereas late-phase behavioral parameters provide strong discriminatory power for imminent expulsion, thereby refining temporal precision. Thus, the structured integration of biologically distinct signals enhances the detection probability and actionable timing. From a management perspective, this stratified alert structure is particularly valuable.

Early notifications allow preparatory observation and staffing allocation, whereas late-phase alerts provide high-confidence warnings for immediate supervision. Such tiered prediction may optimize labor efficiency while maintaining foaling safety. While early-phase indicators improve sensitivity, they may also generate alerts further from foaling, increasing the operational burden for farm staff. In the present study, notifications were observed in 27.8% of mares two days before foaling and 40.9% one day before foaling, although alert frequency remained low during these periods. Similar trade-offs between early detection and increased false alerts have been reported for wearable and activity-based monitoring systems [17,22,23]. This finding highlights the balance between early sensitivity and alert burden. The tiered interpretation proposed here may help mitigate this issue by distinguishing preparatory notifications from high-confidence alerts close to delivery.

Previous studies using accelerometers, tail-mounted sensors, and pose-estimation approaches have reported detection rates ranging from approximately 80% to 95%, often requiring wearable or attached devices [39,40,41]. In contrast, the present system provides comparable detection performance while remaining completely noninvasive and requiring no device attachment. In addition, the proposed approach enables two-stage detection by integrating early physiological indicators and late behavioral signs, allowing both preparatory alerts and high-confidence notifications close to delivery. This feature may reduce maintenance requirements, avoid device displacement, and improve usability under commercial breeding conditions.

### 4.3. Pre-Foaling Notifications and Probability Escalation

Notifications were generated in 27.8% of mares two days before foaling and 40.9% of mares one day before foaling. However, the alert frequency remained limited during these periods. These early notifications likely reflect transient physiological or behavioral fluctuations. Previous studies have documented increased restlessness associated with discomfort, environmental stimuli, or circadian influences in late gestation [16,34]. Importantly, the progressive escalation of the detection probability toward the day of foaling suggests that the model captured cumulative signal intensification rather than isolated behavioral events.

These early notifications were typically isolated events reflecting gradual probability escalation rather than continuous high-probability detection. The transition from intermittent alerts to sustained alerts on the day of foaling suggests progressive behavioral and physiological changes toward parturition. Although early notifications occurred before foaling, the mean number of alerts remained low (1.02 ± 0.25 two days before foaling and 2.00 ± 0.38 one day before foaling), suggesting that alert frequency was limited. In practical farm settings, excessive alerting may lead to alarm fatigue and reduced user confidence; however, the relatively low notification frequency observed in this study indicates that the alert burden may remain manageable. Furthermore, the marked increase in alerts on the day of foaling may help distinguish meaningful notifications from occasional early alerts.

Future refinement may include circadian rhythm modeling, individualized baseline normalization, or dynamic threshold adjustment to further reduce premature alerts while preserving high sensitivity.

### 4.4. Undetected Cases and Biological Variability

Four mares were not detected prior to foaling because the predicted probability did not exceed the predefined 70% threshold. In these individuals, locomotor activity and surface temperature exhibited minimal characteristic changes. Equine studies have documented substantial interindividual variation in pain expression, stress responsiveness, and behavioral reactivity [15,34], which may attenuate detectable external signals in some mares. In addition, rapid labor progression or subdued behavioral expression may limit the magnitude of measurable prepartum changes.

These findings also suggest that a fixed probability threshold may be less suitable for mares with low behavioral expressivity, in which only subtle prepartum changes occur. In such individuals, a fixed threshold may fail to capture meaningful but small increases in activity. Lowering the detection threshold may improve sensitivity for these cases; however, this approach may increase early alerts. Therefore, adaptive or individualized threshold adjustment based on baseline behavior may further improve detection performance while maintaining practical usability. Individual undetected cases were reviewed descriptively; however, no consistent abnormalities such as lameness, atypical posture, behavior, or thermographic artifacts were identified. In these mares, all measured variables showed only subtle prepartum changes, resulting in prediction probabilities remaining below the predefined threshold.

### 4.5. Study Strengths, Practical Relevance, and Limitations

The major strengths of this study include the large sample size, validation across multiple commercial farms, and a completely noninvasive monitoring approach. Unlike vaginal transmitters or wearable devices, the present camera-based system requires no attachment, minimizes handling stress, and permits continuous long-term surveillance without interfering with natural behavior. This study also provides biologically interpretable evidence supporting a two-phase prepartum detection model, offering both applied and mechanistic insights into equine parturition monitoring.

However, monitoring was limited to stalled conditions, and all participating farms were located within a similar regional and management context. Therefore, external validity may be limited, and further validation under different breeds, climates, and pasture-based management systems is required. In addition, environmental temperature fluctuations may influence thermal measurements. Although this limitation can be mitigated by using temperature differentials rather than absolute values, further refinement under extreme environmental conditions is needed.

Locomotor activity was calculated using two-dimensional centroid displacement, which does not account for depth variation due to diagonal camera placement. Therefore, the calculated movement represents relative activity rather than absolute movement distance. However, because the camera position was fixed within each stall, this approach provided a consistent index for detecting temporal changes.

The prediction model was not developed using the present dataset but was based on a previously constructed model derived from independent foaling data reported previously [39]. This approach reduces the risk of optimistic performance estimation; however, further independent validation using additional populations is still warranted.

In addition, repeated measurements were obtained from the same mares, and the temporal data were therefore not fully independent. Although Dunnett’s test was used to compare each time point with baseline, more advanced mixed-effects modeling may provide a more rigorous statistical framework for future studies. Furthermore, assumptions of normality and homogeneity were not formally tested prior to parametric analysis. Because behavioral variables included count-type data, alternative nonparametric or mixed-effects approaches may provide more robust statistical inference in future work.

Behavioral indicators used for foaling prediction may also be influenced by feeding management and time-budget allocation. Feeding systems and environmental constraints are known to affect locomotion, posture transitions, and activity patterns in horses, which may influence automated detection performance. Previous studies have demonstrated that feeding management significantly alters behavioral expression and activity allocation in equines, highlighting the importance of management context when interpreting behavioral monitoring data. These findings suggest that calibration under different feeding systems and management conditions may further improve the robustness of the proposed monitoring approach.

## 5. Conclusions

Thermal imaging combined with model-based image recognition may enable noninvasive detection of foaling-related changes in Thoroughbred mares. The observed temporal patterns in behavioral and thermal indicators suggest a biologically interpretable two-phase prepartum process. The proposed approach showed high detection performance under the present commercial farm conditions and may support preparatory monitoring and timely supervision. However, broader practical deployment will require further validation across breeds, management systems, and environmental conditions, as well as continued refinement of alert thresholds and model transparency.

## Figures and Tables

**Table 1:** Comparison of the foaling detection performance between model 1 and model 2.

Models	Monitored Foalings (n)	Detected Foalings (n)	Missed Detections (n)	Detection Rate (%)	Detection on Foaling Day (%)	Time from First Detection to Foaling (Min, Mean ± SE)
Model 1	115	92	23	80.0	-	186 ± 20
Model 2	115	109	6	94.8	94.8	89 ± 14

Detection rate was calculated as the proportion of detected foalings relative to the total number of monitored foalings. Among detected cases, two mares were identified at the time of foaling, whereas four mares were not detected prior to foaling. Time from first detection to foaling is presented as mean ± standard error (SE).

## Data Availability

The raw data supporting the conclusions of this article will be made available by the authors on request.

## Abbreviations

The following abbreviations are used in this manuscript:

SD	Standard deviation
SE	Standard error
